# The Impact of Opportunistic Salpingectomy on Ovarian Reserve: A Systematic Review

**DOI:** 10.3390/jcm13113296

**Published:** 2024-06-03

**Authors:** Teodora Radu, Matyas Mar, Vlad Tudorache, Claudiu Marginean

**Affiliations:** 1 Department of Obstetrics and Gynecology, “George Emil Palade” University of Medicine, Pharmacy, Sciences and Technology, 540142 Târgu-Mures, Romania; 2 Institution Organizing University Doctoral Studies (IOSUD) “George Emil Palade” University of Medicine, Pharmacy, Sciences and Technology, 540142 Târgu-Mures, Romania

**Keywords:** opportunistic salpingectomy, hysterectomy, ovarian reserve, ovarian cancer

## Abstract

**Background:** In the last decade, increasing evidence has suggested that high-grade serous ovarian cancers may have their origin in the fallopian tube rather than the ovary. This emerging theory presents an opportunity to prevent epithelial ovarian cancer by incorporating prophylactic bilateral salpingectomy into all surgical procedures for average-risk women. The aim of this review is to investigate the hypothesis that bilateral salpingectomy (BS) may have a negative impact on ovarian reserve, not only following hysterectomy for benign uterine pathologies but also when performed during cesarean sections as a method of sterilization or as a treatment for hydrosalpinx in Assisted Reproductive Technology interventions. **Methods:** PubMed, Medline, Google Scholar, and Cochrane were searched for original studies, meta-analyses, and opinion articles published between 2014 and 2024. **Results:** Out of 114 records from the database search, after the removal of duplicates, 102 articles were considered relevant for the current study. **Conclusions:** Performing opportunistic salpingectomy seems to have no adverse impact on ovarian function in the short term. However, because there is an existing risk of damaging ovarian blood supply during salpingectomy, there are concerns about potential long-term adverse effects on the ovarian reserve, which need further investigation.

## 1. Introduction

Among women, ovarian cancer is the fifth leading cause of cancer-related deaths in women and exhibits the highest mortality rate of all gynecologic malignancies. The overall survival rate for epithelial ovarian cancer has improved significantly in the past 50 years [1]. Current efforts to screen for ovarian cancer have proven ineffective, with associated false-positive results, leading to unnecessary surgery and complications associated with surgeries [2].

A hypothesis has been formulated, proposing that the origin of the most frequent type of epithelial ovarian cancer (EOC), high-grade serous carcinoma (HGSC), is the epithelium of the fallopian tube. For this EOC subtype, no precursive lesions have been found in the ovaries; however, a potential precursor for HGSC, known as serous tubal intraepithelial carcinoma (STIC), has been observed in the fimbrial part of the fallopian tube [3]. These STIC lesions are thought to implant on the ovarian or peritoneal surfaces and, over a concealed period, progress into rapidly growing HGSC. Therefore, bilateral salpingectomy at the time of a planned intra-abdominal surgery, an intervention called “opportunistic salpingectomy”, could become the primary prevention method for EOC [4,5].

In the last decade, opportunistic salpingectomy has become the standard of care for EOC risk reduction in women undergoing interventions for benign gynecological pathologies [6]. Furthermore, there is a growing consideration for advocating for salpingectomy over proximal tubal occlusion during sterilization procedures for women who have finished childbearing [7,8]. There are several studies that support the endorsement of bilateral salpingectomy as a contraceptive method in women who desire permanent sterilization at the time of cesarean delivery [9,10].

Another matter of debate is the surgical technique that should be implemented for the treatment of hydrosalpinx before IVF (in vitro fertilization). Proximal tubal occlusion might be a viable alternative to salpingectomy, with the advantages of fewer surgical risks and avoiding the disruption of normal blood flow to the ovary [11].

## 2. Materials and Methods

We conducted a PubMed/Medline/Google Scholar/Cochrane database search in March 2024. We targeted articles published from 2014 to 2024, regarding the effect of bilateral salpingectomy on ovarian reserve during interventions for benign gynecological pathologies. We used controlled vocabulary, more exactly, MeSH terms: “opportunistic” + “salpingectomy” + “ovarian” + “function”, and also entry terms, like: “ovarian” + “reserve” + “bilateral” + “salpingectomy”, “hysterectomy” + “bilateral” + “salpingectomy”. For articles that analyzed the results of bilateral salpingectomy during cesarean sections, we used the following terms: “salpingectomy” + “cesarean section” + “ovarian” + “reserve”; for the articles regarding the effects of salpingectomy on IVF cycle results, we used the following terms: “ART” (Assisted Reproductive Technology) + “after” + “salpingectomy” and “IVF” + “salpingectomy”.

We considered relevant articles that met the following conditions: (1) retrospective or prospective studies, as well as meta-analyses, which assessed reproductive-age women between 30 and 50 years old, who requested a sterilization method, (proximal tubal occlusion orbilateral salpingectomy following cesarean section) or articles that included the same cathegory of patients to whom a tubal intervention for hydrosalpinx was made (2) articles that included perimenopausal patients with bilateral salpingectomy as a method of ovarian cancer prophylaxis during abdominal or laparoscopic hysterectomy; (3) those that evaluated pre/post-operative serum levels of AMH (Anti-Müllerian Hormone), FSH (Follicle-Stimulating Hormone), AFC (Antral Follicle Count), and estradiol, characteristics of the IVF procedure, and their impact on ovarian response during controlled stimulation cycles. Articles in languages other than English and papers without an available full text were excluded. Other exclusion criteria involved articles that did not include preoperativeand postoperativeevaluation and studies that used animals or in vitro models.

The primary objective was to assess whether opportunistic salpingectomy during hysterectomy for benign pathologies of the uterus might have a negative effect on ovarian function and if the type of surgical intervention addressed (laparotomy/laparoscopy) influences post-operative ovarian reserve. Serum Follicle-Stimulating Hormone (FSH), Anti-Müllerian Hormone (AMH), and Antral Follicle Count (AFC) were considered markers for evaluating ovarian reserve. We observed and compared the variations between these markers pre-operatively and post-operatively. The secondary objective was to assess the risks and benefits of salpingectomy at the time of cesarean section as a means to reduce ovarian cancer for women who have chosen to conclude childbearing.

The third objective was to evaluate the potential negative effect of tubal surgery on ovarian response in controlled stimulation cycles.

Given the objectives of this systematic review, the control groups were chosen as follows: The first control group consisted of women who underwent hysterectomy without opportunistic salpingectomy. The second control group included women who had proximal tubal ligation instead of bilateral salpingectomy. The third control group comprised patients who used assisted reproductive techniques and did not have their fallopian tubes removed.

## 3. Results

**Figure 1:**
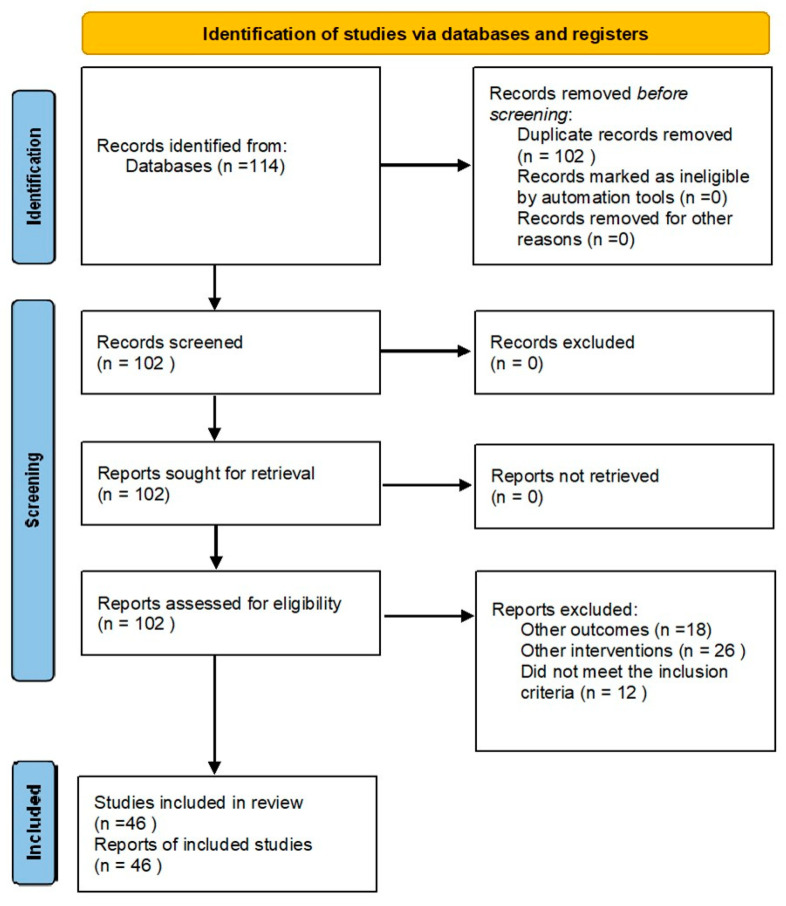
Prisma 2020 flow diagram for new systematic reviews, which included searches of databases and registers only.

Out of 114 records from the database search, after the removal of duplicates, 102 articles were screened for relevance. A total of 56 articles were excluded, with 45 being deemed irrelevant, and 11 studies lacking full-text availability. Therefore, the total number of articles included in the study was 46:29 articles regarding opportunistic salpingectomy during interventions for benign uterine pathologies, 11 articles related to the effects of tubal surgery on ovarian reserve in stimulation cycles within ART, and 6 articles analyzing the impact of bilateral salpingectomy as a means of surgical sterilization during cesarean section (Figure 1). 

Regarding the impact of opportunistic salpingectomy on ovarian function, most studies concluded that there is no relationship between the removal of the fallopian tubes and the impairment of ovarian vascularization. Four studies found an association between opportunistic salpingectomy and its negative impact on ovarian reserve: two studies identified a relationship between changes in AMH and FSH levels at 3 months post-intervention, one study highlighted a decrease in AMH levels after hysterectomy (both abdominal and laparoscopic), and the fourth study concluded that there is a connection between bilateral salpingectomy and decreased AMH levels, as well as a decrease in the AFC (Table 1). 

Of the six articles analyzed on the topic of the impact of bilateral salpingectomy on ovarian function as a means of surgical sterilization during cesarean section, only two were original articles, while the others were reviews. One of the original articles concluded that serum AMH levels were not significantly different 6–8 weeks post-salpingectomy, while the other additionally analyzed the AFC at 3 and 6 months post-operatively and reached the same conclusion.

Two-thirds of the studies on the potential effects of bilateral salpingectomy on ovarian response in controlled stimulation cycles have drawn attention to the need for increased gonadotropin doses and stimulation days, also highlighting lower fertilization rates and a smaller number of grade 1 embryos. Apparently, there are noticeable decreases in serum AMH, as well as in the AFC (Table 2).

## 4. Discussion

Medeiros et al. first introduced the concept of prophylactic salpingectomy for ovarian cancer prevention in 2006, which was strongly recommended in subsequent studies. However, concerns regarding post-surgical ovarian function may influence the decision-making process regarding fallopian tube resection during hysterectomy for benign indications [5,12].

This review draws attention to the lack of a consensus in the specialized literature regarding the impact of opportunistic salpingectomy on ovarian function, as there are several studies demonstrating that this intervention has a negative effect on ovarian reserve. This aspect is most clearly illustrated in patients undergoing assisted human reproduction methods, where the treatment response appears to be delayed in those who have undergone bilateral salpingectomy for hydrosalpinx. Moreover, it seems that fertilization rates are lower compared to those who have not undergone this procedure. Most likely, the lack of consensus regarding ovarian function post-salpingectomy resides in the small sample size of studies and the short post-operative follow-up period, which was often limited to 3–6 months post-operatively. 

### 4.1. Opportunistic Salpingectomy during Hysterectomy for Benign Uterine Pathologies 

In their study on the effect of bilateral salpingectomy on ovarian function, Tehranian et al. found a significantly decreased serum AMH level at 3 months post-operatively in both groups (*p* < 0.001) [33]. This finding is consistent with the outcomes reported by Yuan Z et al. (2019), who identified a decreased post-operative AMH level in patients who underwent hysterectomy with bilateral salpingectomy (*p* < 0.001). Furthermore, they noted an elevated post-operative FSH level in these patients (*p* < 0.001). Limitations of this study include the small sample size (84 patients), having a major surgery, and the short follow-up after hysterectomy (only 6 weeks) [13].

Suneja et al. concurred that bilateral opportunistic salpingectomy during hysterectomy does not seem to have any short-term effects on ovarian function or elevate surgical risk [14]. The same opinion is shared by Rustamov et al. in a large cross-sectional study, which failed to identify any statistically significant difference in AMH levels among women who underwent bilateral salpingectomy compared to those who did not undergo this intervention [15]. Another case–control study from 2021 concluded that this procedure is a safe and convenient treatment and it does not have any deleterious effect on ovarian reserve [16]. There have been other studies with the same objectives that reached similar conclusions; however, the longest follow-up of patients was for a period of 1 year, and the patient cohort enrolled was limited in size [17,18].

Concerning the impact of laparoscopic hysterectomy with bilateral salpingectomy on ovarian function, Wang et al., Findley et al., and Zahra et al. concluded that there is no statistically significant difference between the two groups [19,20,21]. The retrospective study by Wang et al. showed no significant difference between the salpingectomy group and control group at 3 and 9 months after the intervention, regarding AMH, E2, FSH, and LH levels and AFC (all *p* > 0.05). Comparing AMH levels between total abdominal hysterectomy and total laparoscopic hysterectomy, Tavana et al. found a significant decrease in this hormone level after both methods of hysterectomy. However, a lower level of AMH was noted in the total abdominal hysterectomy group [22]. 

### 4.2. Salpingectomy at the Time of Cesarean Section

In a study that aimed to compare longitudinal changes in ovarian reserve markers after cesarean section with or without salpingectomy, Ida T et al. noticed that AMH levels increased over 6 months of follow-up in both groups, but no clinically significant difference was observed (baseline 0.69 ng/mL in the control group vs. 0.49 ng/mL in the salpingectomygroup *p* = 0.64; at 3 months: 1.35 ng/mL vs. 1.45 ng/mL, *p* = 0.79; at 6 months: 1.74 ng/mL vs. 2.60 ng/mL, *p* = 0.27). No difference in the Antral Follicle Count was observed [34]. 

Regarding the optimal sterilization technique during cesarean section, Ganer et al. concluded that bilateral salpingectomy appears to be as safe as tubal ligation, concerning ovarian reserve and intra/post-operative complications [8]. AMH serum levels were not significantly different between the groups 6–8 weeks following surgery. As salpingectomy has the advantage of reducing the risk of ovarian cancer, it could be suggested to patients planning elective cesarean sections [35].

On the other hand, according to Vignarajan et al., PTO (proximal tubal occlusion) is a better surgical technique. In their randomized controlled trial, they observed a notable decline in ovarian reserve parameters following bilateral salpingectomy, with both AMH levels and AFC experiencing a significant decrease (*p* < 0.001) [8]. A similar conclusion was drawn by three other studies. PTO involved a higher fertilization rate compared to salpingectomy in the treatment of hydrosalpinx in patients before undergoing IVF [36,37,38]. Additionally, both salpingectomy and PTO effectively eliminated the retrograde flow of the toxic hydrosalpinx fluid into the uterine cavity. This resulted in improved access to the ovary, optimalconditions for oocyte retrieval, increased endometrial receptivity, and facilitation of fertilization and pregnancy [11,12,39,40].

Furthermore, salpingectomy performed after ectopic pregnancy in women requiring future ART results in a reduced number of oocytes from the operated adnexa. However, the overall reproductive outcomes do not show a statistically significant difference compared to women who have not undergone salpingectomy [41].

### 4.3. The Impact of Tubal Surgery on Ovarian Response during Controlled Stimulation Cycles

Xu-ping et al. retrospectively compared serum AMH levels measured on the ovulation induction day in patients with unilateral, bilateral, and no tubal surgery and found a mean AMH level significantly higher in women without tubal surgery, compared to those with bilateral salpingectomy. Also, the FSH level was higher in the group with bilateral salpingectomy with a *p*-value = 0.048 [7]. Furthermore, Jacob GP et al. concluded in their study that salpingectomy performed after ectopic pregnancy in women requiring future ART results in a reduced number of oocytes from the operated adnexa. However, the outcomes regarding the live births did not show a statistically significant difference compared to women who had not undergone salpingectomy [42]. 

In a meta-analysis aiming to test the hypothesis that salpingectomy could compromise ovarian function, Mohamed et al. found eight eligible studies (cross-sectional and randomized controlled trials) in which serum AMH and FSH, as well as the AFC, were analyzed post-hysterectomy, myomectomy, and sterilization. Their study found no short-term significant changes in serum AMH but revealed a lower AFC in the salpingectomy group compared to the control group. The limitation of the meta-analysis was the very small number of studies included: only four studies involved salpingectomy during hysterectomy/myomectomy or sterilization, three studies evaluated the impact of salpingectomy on ovarian reserve after ectopic pregnancy, and one study assessed the same impact after salpingectomy for tubal pathology [38].

In another meta-analysis that included five studies, comprising 648 patients, a comparative analysis of AMH, FSH values, and AFC was conducted between patients who underwent proximal tubal occlusion and salpingectomy for treating hydrosalpinx, aiming to evaluate pregnancy rates in ART [43]. The Follicle-Stimulating Hormone values did not differ between the groups, while AMH values and the AFC were significantly higher in the salpingectomy group compared to the proximal tubal occlusion group. Therefore, Shuxie et al. [43] concluded that in the short term, salpingectomy affected ovarian reserve more than proximal tubal occlusion. They found no significant difference in FSH levels between the two techniques, proximal tubal occlusion and laparoscopic salpingectomy, but compared to the salpingectomy group, the PTO group had a significantly higher AFC, both in the 2-month subgroup and overall. Additionally, the PTO group showed significantly higher AMH levels in each specific time subgroup as well as overall.

Although several individual studies with a limited number of cases have demonstrated variable outcomes, a meta-analysis conducted by Mohamed AA et al. in 2017, indicated that salpingectomy has no adverse effects on ovarian reserve [38].

## 5. Conclusions

Regarding bilateral salpingectomy during hysterectomy for benign pathologies of the uterus, most studies have not identified any short-term impairment of ovarian function, which is most commonly demonstrated through serum AMH measurement. However, our study identified a lack of consensus concerning the impact on ovarian vascularization following bilateral salpingectomy, as there are some studies cited that show contrary results. As for bilateral salpingectomy as a means of sterilization during cesarean section, the study results are also divergent. Some studies did not identify a statistically significant correlation between this procedure and post-salpingectomy ovarian function, while others, comparing bilateral salpingectomy with proximal tubal occlusion, found a notable decrease in ovarian parameters in the salpingectomy groups, compared to patients who underwent tubal occlusion. The influence of bilateral salpingectomy on ovarian reserve in patients who used assisted human reproduction techniques did not have a major impact. Most studies suggest that this procedure does not negatively affect the outcomes of in vitro fertilization cycles; however, some report that bilateral salpingectomy influenced the number of oocytes retrieved, but not the number of pregnancies achieved.

To conclude, opportunistic salpingectomy seems to have no short-term effect on ovarian function. However, because there is a potential risk of damaging ovarian blood supply during salpingectomy, there is a concern about potential long-term adverse effects on ovarian reserve that need further investigation.

## Figures and Tables

**Table 1:** Opportunistic salpingectomy during hysterectomy for benign uterine pathologies.

Study Characteristics	Study Design	Indication	Intervention	Findings
Behnamfar et al. [5], 2017	Randomized controlled trial	Benign pathologies of the uterus in premenopause	Abdominal hysterectomy +/− BS	Mean FSH, LH did not Differ significantly at 6 months postoperatively
Nassif et al. [12], 2020	Randomized controlled trial	Benign pathologies	Vaginal hysterectomy	AMH, FSH, AFC, FI, VI, VFI, OvAge No statistically significant differences at 6 and 8 months postoperatively
Yuan et al. [13], 2018	Prospective longitudinal study	Uterine myoma/Early stage cervical cancer	Laparoscopic/ abdominal hysterectomy with BS	AMH, FSH AMH levels lower and FSH levels higher at 1 week and 6 weeks postoperatively
Suneja et al. [14], 2020	Observational	Benign pathologies	Abdominal/laparoscopic/vaginal hysterectomy +/− BS	AMH, ovarian Doppler indices(RI, PI, S/Dratio) No significant differences in AMH levels or Doppler indices 3 months post-operatively
Rustamov et al. [15], 2016	Retrospective cross-sectional	Benign pathologies	Abdominal salpingectomy, salpingo-oophorectomy, cystectomy, excision of endometrioma	AMH, AFC, FSH No significant differerence in their levels post-operatively
Gareeb et al. [16], 2021	Case control	Benign uterine disease	Abdominal hysterectomy +/− BS	FSH, AFC, ovarian volume, RI, PI ovarian artery No significant differences postoperatively or between the groups
Abdelazim et al. [17], 2015	Prospective	Benign uterine pathology	Abdominal hysterectomy	AMH, FSH, E2, ovarian volume Statistically insignifiant differences in levels/volume at 6 and 12 months after surgery
Poonam et al. [18], 2020	Randomized controlled trial	Benign pathologies	Abdominal hysterectomy +/− BS	FSH, LH, E2 BS did not have any negative effect on ovarian function
Wang et al. [19], 2021	Randomized controlled trial	Benign uterine diseases in premenopausal women	Laparoscopic hysterectomy +/− OS	AMH, FSH, LH, E2, AFC No differences between the groups at 3 and 9 months postoperatively
Findley et al. [20], 2014	Randomized controlled trial	Benign uterine pathologies	Laparoscopic hysterectomy	No difference in AMH levels 4 to 6 weeks and 3 months postoperatively
Asgari et al. [21], 2018	Randomized controlled trial	Abnormal uterine bleeding related to benign pathology	Total laparoscopic hysterectomy +/− BS	AMH, FSH Significant lower level of AMH and higher level of fSH at 3 months postoperatively
Tavana et al. [22], 2021	Prospective	Abnormal uterine bleeding without anatomical or hormonal reasons	Total abdominal hysterectomy (TAH) compared with Total laparoscopic hysterectomy (TLH)	AMH levels decreased after both methods, the decrease was greater in TAH
Venturella et al. [23], 2016	Observational study	Abnormal uterine bleeding (benign pathologies)	Total laparoscopic hysterectomy + prophylactic BS	AMH, FSH, AFC, OvAge Without any negative effect on ovarian reserve 3 to 5 years after surgery
Naaman et al. [24], 2016	Open-label, prospective cohort	Benign uterine pathologies	Abdominal hysterectomy + BS/Fimbriectomy	FSH, AMH, S/D ratio, RI ovarian artery No significant differences between and 6 months after surgery/between groups
Venturella et al. [25], 2015	Randomized controlled trial	Uterine myoma and tubal surgical sterilization	Laparoscopic hysterectomy+BS/BS	AMH, FSH, AFC, VI, FI, VFI Standard vs. wide resection—no difference in ovarian reserve or vascular flow parameters
Singh et al. [26], 2023	Prospective case control	Benign pathologies	Abdominal hysterectomy +/−BS	AMH, FSH, LH No statistical significance impact on ovarian reserve
Atalay et al. [27], 2016	Prospective longitudinal	Benign uterine disorders	TLH + BS vs. TAH + BS	AMH, FSH, LH, E2, inhibin B, ovarian volume AMH and ovarian volume decreased significantly 6 months postoperatively in the TAH-BS group

**Table 2:** Studies evaluating response in controlled stimulation cycles and impact on ovarian reserve after salpingectomy.

Study Characteristics (Year)	Study Design	Indication	Intervention	Findings
Ye et al. [7] 2015	Retrospective cohort study	Tuboovarian Abscess Ectopic Pregnancy Hydrosalpinx	Unilateral/Bilateral Salpingectomy	AMH levels lower in the bilateral salpingectomy group (183.48 vs. 127.11, *p* < 0.037), FSH levels higher in the same group (9.13 vs. 7.85, *p* = 0.048)
Vignarajan et al. [11] 2018	Randomized controlled trial	Hydrosalpinx	Bilateral Salpingectomy/ Proximal Tubal Occlusion	Significant fall in AMH of the salpingectomy group (3.7 vs. 2.6, *p* < 0.001) and AFC (10.6 vs. 8.6, *p* < 0.001) Salpingectomy group required higher doses of gonadotropines and more days of stimulation + lower fertilization rates and lower number of grade 1 embryos
Huang et al. [28] 2019	Retrospective cohort study	Hydrosalpinx	Laparoscopic Salpingectomy	No significant change in AMH, FSH, E2 levels 3 months after surgery
Reitz et al. [29] 2023	Case-control study	Ectopic Pregnancy Hydrosalpinx	Unilateral Salpingectomy	Mean number of mature follicles significantly reduced after salpingectomy (3.00 vs. 5.08, *p* = 0.048)
Ho Cheng-Yu et al. [30] 2022	Retrospective case-control study	Hydrosalpinx Ectopic Pregnancy	Unilateral/Bilateral Salpingectomy	AFC and AMH levels statistically significant lower in the salpingectomy group The number of oocytes retrived significantly lower in the same group (10.4 +/− 5.2 vs. 12.2 +/− 3.8, *p* = 0.06)
Yilei H et al. [31] 2023	Randomized controlled trial	Hydrosalpinx	Bilateral Salpingectomy	Higher levels of basal FSH in the salpingectomy group and lower AMH levels (*p <* 0.05)
Gluck et al. [32] 2018	Retrospective cohort study	Hydrosalpinx Ectopic Pregnancy	Unilateral/Bilateral Salpingectomy	AMH, FSH, E2, Progesterone levels not significantly different in the groups AFC, oocytes retrieved, amount of Gonadotropin used and number of embryos transferred not significantly different

## Data Availability

Data is contained within the article.

## Abbreviations

The following abbreviations are used in this manuscript:

BS	Bilateral salpingectomy
OS	Opportunistic salpingectomy
EOC	Epithelial ovarian cancer
HGSC	High-grade squamous cell
STIC	Serous tubal intraepithelial carcinoma
IVF	In Vitro Fertilization
ART	Assisted Reproductive Technology
AMH	Anti-Müllerian Hormone
FSH	Follicle-Stimulating Hormone
LH	Luteinizing Hormone
AFC	Antral Follicle Count
C-section	Cesarean section
PTO	Proximal tubal occlusion
E2	Estradiol
OvAge	Ovarian age
RI	Ovarian artery resistance index
PI	Ovarian artery pulsatility index
S/D	Systolic/diastolic ratio ovarian artery
